# Investigating the efficacy and safety of calcipotriol/betamethasone dipropionate foam and laser microporation for psoriatic nail disease—A hybrid trial using a smartphone application, optical coherence tomography, and patient‐reported outcome measures

**DOI:** 10.1111/dth.15965

**Published:** 2022-11-23

**Authors:** Vinzent Kevin Ortner, Victor Desmond Mandel, Kresten Skak, John Robert Zibert, Mélanie Bourlioux, Christoffer Valdemar Nissen, Christine Sofie Krohn Fuchs, Peter Alshede Philipsen, Merete Haedersdal

**Affiliations:** ^1^ Department of Dermatology Copenhagen University Hospital, Bispebjerg and Frederiksberg Copenhagen Denmark; ^2^ LEO Pharma A/S Ballerup Denmark; ^3^ Dermatology Unit, Surgical, Medical and Dental Department of Morphological Sciences Related to Transplant, Oncology and Regenerative Medicine University of Modena and Reggio Emilia Modena Italy; ^4^ Porphyria and Rare Diseases Unit San Gallicano Dermatological Institute – IRCCS Rome Italy; ^5^ Future‐Brain Aps Copenhagen Denmark; ^6^ Omhu A/S LEO Innovation Lab Copenhagen Denmark

**Keywords:** drug delivery, imaging, nail disease, psoriasis, smartphone, topical

## Abstract

There is a lack of efficacious topical treatments for patients suffering from psoriatic nail disease (PND). We investigated the efficacy of Calcipotriol‐Betamethasone Dipropionate (Cal/BD) foam with and without ablative fractional laser (AFL) in patients with PND. A total of 144 nails from 11 patients were treated in a 24‐week long, open‐label, randomized, intra‐patient controlled proof‐of‐concept hybrid trial. In addition to daily Cal/BD foam application, half of each patient's psoriatic nails were randomized to receive optical coherence tomography (OCT)‐guided AFL treatment at baseline, 6‐, and 12‐week follow‐ups. *In‐clinic* assessment (N‐NAIL), patient‐reported outcomes (PROMs), and drug consumption were supplemented by *remote* evaluation of 15 subclinical OCT features, smartphone app‐based safety monitoring, and photo‐based assessment (NAPSI). After 24 weeks of Cal/BD foam treatment, patients achieved a significant improvement (*p* < 0.001) in both clinical (N‐NAIL −76%, NAPSI −68%) and subclinical (OCT −43%) PND severity as well as a 71% reduction in PROMs. AFL‐assisted Cal/BD treatment led to higher clinical (N‐NAIL −85%, NAPSI −78%) and OCT‐assessed (−46%) reduction of PND signs than Cal/BD alone (N‐NAIL −66%, NAPSI −58%, OCT −37%), but did not reach statistical significance. Smartphone app images documented adverse events and mild local skin reactions, particularly erythema (75%), laser‐induced swelling (28%), and crusting (27%). This hybrid trial demonstrated a reduction in clinical NAPSI and N‐NAIL scores, subclinical OCT features, and PROMs, suggesting that Cal/BD foam is a safe and efficacious treatment for PND. Larger trials are warranted to prove the clinical benefit of AFL pretreatment as a Cal/BD delivery enhancer.

## 1 INTRODUCTION

Psoriatic nail disease (PND) affects more than 50% of cutaneous psoriasis and 80% psoriatic arthritis patients and is considered a difficult‐to‐treat condition.^1^, ^2^ In the past 20 years, 31 clinical trials on topical treatment of PND have been conducted (Table 1), with corticosteroids (*n* = 16, clobetasol, betamethasone) and Vitamin D derivatives (*n* = 13, calcipotriol, tacalcitol) remaining the most studied agents.^3^, ^4^, ^5^, ^6^, ^7^, ^8^, ^9^, ^10^, ^11^, ^12^, ^13^, ^14^, ^15^, ^16^, ^17^, ^18^, ^19^, ^20^, ^21^, ^22^, ^23^, ^24^, ^25^, ^26^, ^27^, ^28^, ^29^, ^30^, ^31^, ^32^, ^33^ Products containing both calcipotriol and betamethasone dipropionate (Cal/BD) are highly effective against psoriasis and have become available as a hypersaturated aerosol foam. In addition to superior efficacy in treating skin lesions, results of a 12‐week randomized controlled trial (RCT) on Cal/BD foam for PND on fingernails showed a 44% decrease of clinical symptoms with only minor side effects.^4^

**TABLE 1:** Overview of clinical trials (2001–2021) on topical treatments for psoriatic nail disease

Year/author	Study design	# of patients (nails)	Additional intervention	Topical	Time frame	Formulation	Frequency	Clinical results	Tolerability	Toenails included
2021/Essa Abd Elazim et al^33^	RCT (vs. topical only)	27 (−)	CO_2_	Tazarotene	6 months	Gel (0.1%)	Daily/3 months	mNAPSI baseline 12.6, EOT 4.9 (−48%)	laser‐induced bleeding, erythema, and pain	No
2021/Shehadeh et al^3^	RCT (vs. untreated)	17 (−)	PDL + CO_2_	Cal/BD	11 months	Gel	daily/3 months	NAPSI baseline 21.4, EOT 14.1 (−34%)	Irritation, erythema, purpura, pain (VAS 5.7)	No
2020/Gregoriou et al.^4^	RCT (vs. PDL)	16 (110)	–	Cal/BD	12 weeks	Foam	Daily/12 weeks	Mean NAPSI baseline 7.9, EOT 4.4 (−44%)	Irritation	No
2020/Tehranchinia et al^5^	RCT (vs. ALA‐PD)	8 (69)	Occlusion	CP	24 weeks	Ointment (0.05%)	Daily for 15 weeks	NAPSI baseline 6.0, EOT 3.9 (35%)	–	No
2019/Boontaveeyuwat et al.^6^	RCT (vs. injection)	16 (48)	–	CP	6 months	Ointment (0.05%)	Twice daily/6 months	tNAPSI baseline 5.7, EOT 5.0 (−12%)	Focal erythematous atrophy (*n* = 1), focal hypopigmentation and telangiectasia (*n* = 1), pain	No
2018/Brandi et al.^7^	Uncontrolled	15 (−)	–	CP	24 weeks	Solution (0.05%)	Daily/4–6 months	Mean NAPSI baseline 6.1, EOT 2.6 (−57%)	No side effects	–
2018/Saki et al.^8^	RCT	16 (–)	(a) Iontophoresis	(a) TA (b) Cal/BD	6 months	(a) Solution (b) Ointment	(a) Monthly/6 months (b) daily/6 months	NAPSI (a) baseline 12.5, EOT 3.8 (−70%) (b) baseline 11.9, EOT 5.3 (−56%)	–	No
2018/Piraccini et al.^9^	Uncontrolled	34 (34)	–	K101‐03	8 weeks	Solution	Daily/8 weeks	94% of patients reported improved appearance	–	–
2017/Arango‐Duque et al.^10^	RCT	11 (–)	(a) PDL + topical (b) Nd:YAG + topical	Cal/BD	3 months	Gel	Daily/1 week after monthly laser treatment	NAPSI baseline 34.9, EOT 12.5 (−46%)	Laser‐related petechiae	No
2017/Piraccini et al.^11^	Uncontrolled	34 (34)	–	K101‐03	8 weeks	Solution	Daily/8 weeks	94.1% showed improvement	–	–
2015/Lin et al.^15^	RCT	28 (–)	–	(a) IN (b) Cal	24 weeks	(a) Oil (b) solution (50 μg/ml)	Twice daily/24 weeks	shNAPSI (a) −51% (b) −27%	Irritation (a) *n* = 2 (b) *n* = 10	No
2014/Cantoresi et al.^13^	RCT (vs. vehicle)	81 (810)	–	HCPC	24 weeks	Lacquer	Once daily for 24 weeks	67.7% of patients mNAPSI <4 at EOT (placebo 40.6%)	No adverse reactions	No
2014/Lin et al.^20^	RCT (vs. vehicle)	27 (291)	–	IN	24 weeks	Oil	Twice daily for 24 weeks	shNAPSI/mtNAPSI −49.8%/−59.3% after 12 weeks	No side effects	No
2014/Kole et al.^14^	RCT	10 (12)	–	(a) Calcitriol (b) BD	20 weeks	(a) ointment (3 μg/g) (b) ointment (64 mg/g)	Twice daily/20 weeks	Thickness reduction (−38% vs. −35%), PGA reduction (−50% vs. 7.4%)	No side effects	Yes
2013/Huang^16^	RCT	19 (–)	(a) PDL + topical	Tazarotene	6 months	Cream	–	32% had ≥75% improvement at 6 months	Laser‐related blister	No
2013/De Simone et al.^17^	RCT (vs. untreated)	21 (171)	–	Tacrolimus	12 weeks	Ointment (0.1%)	Daily for 12 weeks	Mean mNAPSI baseline 13.2, EOT 4.6 (−65%), mean NAPSI baseline 23, EOT 10 (−57%)	Acute paronychia	No
2012/Nakamura et al.^18^	RCT (vs. untreated)	15 (–)	–	CP	16 weeks	(a) lacquer (0.05%) (b) lacquer (1%) (c) lacquer (8%)	Twice weekly/16 weeks	mNAPSI baseline 14.3, EOT 6.6 (−52%)	No side effects	–
2012/Fischer‐Levancini et al.^19^	Uncontrolled	6 (–)	Occlusion	Tazarotene	6 months	Ointment (0.1%)	Daily for 6 months	Mean NAPSI baseline 14.3, EOT 2.3 (−88%)	No side effects	–
2011/Lin et al.^12^	Uncontrolled	28 (254)	–	IN	24 weeks	Oil	Twice daily for 24 weeks	NAPSI baseline 36.1, EOT 14.9 (−59%); tNAPSI baseline 11.7, EOT 3.6 (−69%)	Mild irritation (n = 1)	No
2009/Márquez Balbás et al.^21^	Uncontrolled	15 (–)	Occlusion	TM	6 months	Ointment (4 μg/g)	Daily for 6 months	Significant improvement of NAPSI score	No side effects	–
2009/Rigopoulos et al.^22^	Uncontrolled	22 (114)	–	Cal/BD	12 weeks	Ointment	Daily for 12 weeks	Mean NAPSI baseline 5.8, EOT 1.6 (−72%)	Irritation (*n* = 2)	No
2009/Cantoresi et al.^23^	RCT (vs. untreated)	28 (–)	–	HCPC	24 weeks	Lacquer	Daily for 24 weeks	NAPSI baseline 2.8, EOT 1 (−64%)	No side effects	No
2008/Tzung et al.^24^	RCT	24 (–)	–	a) Cal b) Cal/BD	12 weeks	(a) Ointment (0.005%) (b) ointment	(a) twice daily (b) once daily for 12 weeks	53% of patients showed at least moderate improvement after 12 weeks (IGA)	No adverse events	No
2008/Sánchez Regaña et al.^25^	uncontrolled	15 (–)	–	a) CP + b) TM	24 weeks	(a) lacquer (8%) (b) ointment (4 μg/g)	(a) weekly/6 months and (b) 5 times weekly/6 months	mtNAPSI ‐78% between baseline and EOT	No side effects	No
2007/Rigopoulos et al.^26^	RCT	30 (87)	Occlusion	a) Tazarotene b) CP	24 weeks	(a) cream (0.1%) (b) cream (0.05%)	Daily for 12 weeks	Average NAPSI reduction −68% (CPl) and − 75% (Tazarotene) at EOT	(a) Desquamation, erythema of nail fold, periungual irritation, paronychia, irritation *n* = 3) (b) burning sensation (*n* = 1)	Yes
2005/Zakeri et al.^27^	uncontrolled	18 (–)	–	Cal	5 months	Cream (50 μg/g)	Twice daily for 3–5 months	14 patients showed improvement	Periungual irritation, inflammation, irritation, pruritus, oozing (*n* = 2)	No
2005/Sánchez Regaña et al.^28^	uncontrolled	10 (–)	–	CP	9 months	Lacquer (8%)	Daily for 21 days, twice weekly for 9 months	9/10 patients showed good response	No side effects	No
2004/Feliciani et al.^29^	RCT (vs. no topical)	33 (–)	Oral Cyc (3.5 mg/kg/day) and occlusion	Cal	9 months	Cream (50 μg/g)	Twice daily for 3 months	79% improvement by patient and dermatologist after 3 months	No serious side effects	No
2003/Cannavò et al.^30^	RCT (vs. vehicle)	16 (–)	–	Cyc	20 weeks	Oil (70%)	Twice daily for 12 weeks	77% mean improvement after 12 weeks	No side effects	No
2002/Rigopoulos et al.^31^	uncontrolled	62 (251)	–	a) Cal b) CP	12 months	(a) Cream (b) Cream	(a) Five times per week for 6 months AND (b) Twice per week for 12 months	reduction in hyperkeratosis by 81% (fingers) and 73% (toes) at EOT	burning sensation (*n* = 2)	Yes
2001/Scher et al.^32^	RCT (vs. vehicle)	31 (62)	Occlusion	Tazarotene 0.1% (vs. vehicle)	24 weeks	Gel (0.1%)	Daily for 24 weeks	Significant improvement of pitting and onycholysis	mild to moderate AE (*n* = 5, peeling and erythema of proximal nail fold, irritation, paronychia)	No

Abbreviations: BD, betamethasone dipropionate; Cal/BD, calcipotriol/betamethasone dipropionate; Cal, calcipotriol; CO_2_, carbon dioxide laser; CP, clobetasol proprionate; Cyc, cyclosporine; HCPC, hydroxypropyl‐chitosan; IN, indigo naturals (“Lindi oil”; 3.15% indigo blue and 0.15% indirubin extracts in olive oil); K101‐03, compound consisting of propylene glycol, urea, and lactic acid; mtNAPSI, modified tNAPSI; NAPSI, nail psoriasis severity index; Nd:YAG, neodymium‐doped yttrium aluminum garnet; PDL, pulsed dye laser; PGA, physician global assessment (0–3); shNAPSI, single‐hand NAPSI; TA, triamcinolone acetonide; TM, tacalcitol monohydrate; tNAPSI, target NAPSI.

An emerging concept in topical treatment of nail disease is the use of ablative fractional laser (AFL) as a pretreatment to boost the uptake of topically applied drugs.^34^, ^35^ Commonly referred to as laser‐assisted drug delivery, this technique has been established in the management of various skin conditions and gradually expanding into onychology.^36^, ^37^, ^38^ In the context of PND, two RCTs recently investigated the value of AFL as a delivery enhancement strategy for topical treatment using Cal/BD gel and Tazarotene gel, achieving a change from baseline severity between −34% and −48%, respectively.^3^, ^33^

Optical coherence tomography (OCT) is a noninvasive technique that has been used to support clinical assessments of dermatological conditions, including PND.^39^, ^40^ In clinical research, PND treatment response is frequently assessed using clinical scoring systems, such as the Nail Psoriasis Severity Index (NAPSI) and the Nijmegen‐Nail psoriasis Activity Index tool (N‐NAIL).^41^ Using previously reported features from four imaging studies on PND, this study included OCT scans to support clinical outcomes based on N‐NAIL and NAPSI and visualize laser‐tissue interactions.

This proof‐of‐concept study was conceptualized as a hybrid trial, integrating decentralized elements such as patient‐obtained smartphone pictures into a conventional *in‐clinic* setting to enable access to *remote* data collection, study design flexibility, and cross‐validation of outcomes.^42^ Using a blend of *in‐clinic* and *remote* assessments, we aimed to evaluate the clinical and OCT‐assessed subclinical effects, safety, drug consumption, and patient‐reported outcome measures (PROMs) of daily Cal/BD foam for 24 weeks with and without laser pretreatment in patients with PND.

## 2 MATERIALS AND METHODS

### 2.1 Study design

**FIGURE 1:**
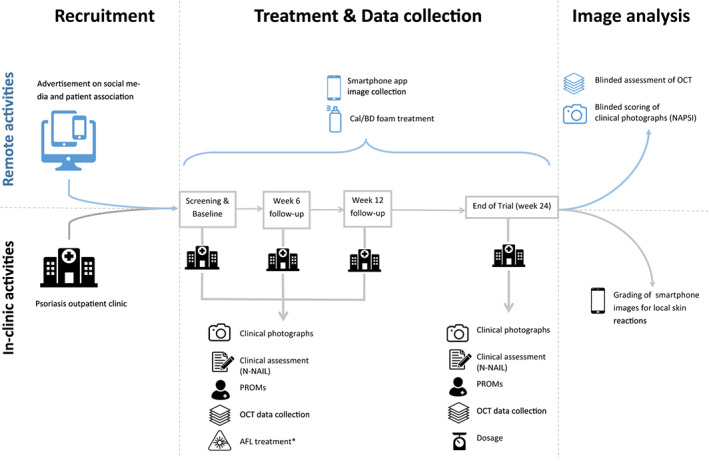
Graphic overview of remote (blue) and in‐clinic (gray) trial workflows during the recruitment, treatment and data collection, and image analysis phase

This is a proof‐of‐concept, single‐center, prospective, open‐label hybrid trial with a randomized intra‐individual comparison of Cal/BD treatment of PND with and without AFL pretreatment. The trial was approved by the Danish Medicines Agency (EudraCT 2019‐002960‐29), the Danish Ethics committee (79048/H‐19047222), the Danish Data Protection Agency (P‐2020‐500), and registered on clinicaltials.gov (NCT04580537). The study was conducted at the Department of Dermatology of the Copenhagen University Hospital, Bispebjerg, Denmark, and monitored by the GCP unit of the Copenhagen University Hospital, Frederiksberg, Denmark. Figure 1 contains a graphical overview of the study design covering all *in‐clinic* and *remote* workflows, which are further described in Table 2.

**TABLE 2:** Overview of in‐clinic and remote components of the hybrid design of the clinical trial

Clinical trial workflows		In‐clinic	Remote
Recruitment method	In‐clinic, social media, patient association	X	X
Available patient population	All of Denmark but required a minimum of four in‐clinic visits	X	X
Pre‐screening	Telephone calls and video consultations	X	X
Study site	Single physical site (Copenhagen University Hospital, Bispebjerg)	X	
Patient visits	In‐clinic treatment visits, virtual photograph‐based follow‐ups	X	X
Informed consent	In‐clinic	X	
Trial activities (e.g., education)	In‐clinic patient education and study app training by treating physician and study nurse	X	
Physical examination	In‐clinic nail psoriasis grading by treating physician	X	
Laboratory testing	In‐clinic mycological testing to exclude nail fungus	X	
Imaging	Standardized clinical photographs collected in‐clinic for remote blinded assessment	X	X
	Smartphone photographs collected remotely by patients for in‐clinic assessment	X	X
	Optical coherence tomography images collected in‐clinic for remote blinded assessment	X	X
Data collection	Collected by study team and smartphone app	X	X
Dispensing of medications	In‐clinic dispensation of Cal/BD foam containers	X	
Outcomes	Collected in‐clinic as well as remote assessors	X	X
Study setup	Single study site, study team comprises in‐clinic staff (project nurse, treating physician) as well as remote assessor	X	X

*Source*: Adapted from Reference 53.

### 2.2 Trial population

Prior to enrollment, all patients gave their informed consent. In addition to *in‐clinic* visits at baseline, after 6, 12, and 24 weeks at the end of the trial (EOT), patients also agreed to the use of a smartphone application for *remote* safety check‐ups.

*Inclusion criteria* were at least 18 years of age, confirmed diagnosis of psoriatic disease, and clinical signs of PND on at least two digits defined as a minimum nail involvement of N‐NAIL score of ≥2 for at least two individual nails. *Exclusion criteria* included pregnant and lactating women, preexisting clinical manifestations of long‐term side effects of corticosteroid use, presence of any skin condition or coloration that would interfere with the evaluation of the clinical response, any non‐psoriatic disease activity within test areas, known allergy to any of the components of Cal/BD foam, artificial nail enhancement or damages associated with it and recent changes in anti‐psoriatic medication. All patients underwent testing for pregnancy and fungal nail disease. Fungal nail disease was excluded using direct KOH microscopy for initial inclusion, followed by confirmatory culture testing. At the EOT visit, patients repeated testing to ensure that no concomitant fungal infection had occurred while enrolled.

### 2.3 Interventions

For even distribution of nail severity per treatment arm, a randomization procedure was used based on N‐NAIL‐score ranking of all nails per patient. An opaque envelope containing one of two randomization codes, “*even*” or “*odd*,” was prepared, signifying either odd or even‐numbered nails to receive AFL pretreatment in addition to Cal/BD foam.

#### 2.3.1 Ablative fractional CO_2_ laser pretreatment

Pretreatment with an ablative fractional carbon‐dioxide laser (DeepFX, UltraPulse® CO_2_, Lumenis; λ = 10,600 nm) was used to create a grid of micropores to promote the delivery of Cal/BD. A topical anesthetic (Pliapel [70 mg/g Lidocaine +70 mg/g Tetracaine], Galderma Nordic AB, Sweden) was applied under occlusion 30 min before laser treatment. Treatment areas were cleaned, disinfected, and patted dry before pre‐cooling with an air cooler (Zimmerman) for 1–2 min.

A fixed density of 10% and a small rectangular spot size were chosen. The nail plates were only treated at baseline using pulse energy settings based on in vivo OCT measurements, following a simple schematic that increased laser energy by 10 mJ/mb for 100 μm of OCT‐measured nail plate thickness (e.g., a 500 μm thick nail received 50 mJ/mb). The lateral and proximal nail folds were treated at baseline and at weeks 6 and 12 with a fixed pulse energy of 40 mJ/mb.

#### 2.3.2 Calcipotriol/betamethasone dipropionate aerosol foam

Participants applied Cal/BD foam (Enstilar®, LEO Pharma A/S) once daily at nighttime (Week 1 to Week 24), to affected nails only. Patients were instructed to apply the foam directly on the included nails and distribute it to cover the nail plate, the nail folds, and the surrounding skin. After applying the product in a thin, even layer, patients were instructed to keep test sites uncovered for at least 15 min to avoid unintentional foam removal. Patients received daily treatment reminders via text message to ensure treatment adherence. Cal/BD foam containers were weighed at EOT to monitor drug consumption and estimate dosing differences.

### 2.4 Assessment of outcome measures

Data collection and outcome assessment were conducted both *in‐clinic* and *remotely*. An overview of all assessed outcome measures is presented in Figure 1.

Fully *in‐clinic* assessed outcome measures comprised clinical efficacy using N‐NAIL and PROMs. OCT scans and clinical photographs were collected *in‐clinic* for a “store‐and‐forward” *remote* image analysis to assess clinical (NAPSI) and subclinical efficacy (OCT). Patient‐obtained smartphone pictures were collected *remotely* for assessment of local skin reactions performed *in‐clinic*, daily for the first week, and weekly until EOT.

#### 2.4.1 Clinical efficacy scoring

Patients were assessed *in‐clinic* by the treating physician using N‐NAIL at all study visits. The N‐NAIL score was used to score all 20 nails using five features (“*onycholysis/oil‐drop discoloration*,” “*pitting*,” “*crumbling*,” “*Beau lines*,” “*subungual hyperkeratosis*”), each ranging from 0 to 3; the highest possible score is 15 per nail and 300 per patient. The minimum score for study participation at baseline was 4 (2 nails scoring 2 or higher).

Using the NAPSI based on clinical photographs of hands and feet captured in‐clinic by a study nurse, PND severity was assessed remotely by a blinded board‐certified dermatologist (VDM).^41^, ^43^ Features are assessed per quadrant and categorized as either nail matrix (“*pitting*,” “*leukonychia*,” “*red spots in the lunula*,” “*crumbling*”) or nail bed psoriasis (“*onycholysis*,” “*oil drop discoloration*,” “*splinter hemorrhages*,” “*subungual hyperkeratosis*”). The total score per nail can range from 0 to 8, with a maximum of 160 points per patient.

#### 2.4.2 Patient‐reported outcome measures

At *in‐clinic* visits, patient‐reported outcome measures (PROMs), consisting of two questionnaires, were assessed: a modified version of the DLQI (Dermatology Life Quality Index) adapted to assess the PND burden and a set of questions concerning self‐evaluated nail disease severity, Cal/BD‐related discomfort, treatment preference, and AFL‐related pain.

The modified Danish version of the Dermatology Life Quality Index (mDLQI) consists of 10 questions rated from 0 to 3 (maximum score: 30; see Table S1). The set of questions used a numerical scale ranging from 0 to 10 to evaluate disease severity, Cal/BD treatment discomfort, and AFL‐pain where “0” would correspond to “*currently disease‐free*”/“*no discomfort/no pain*” and “10” to “*extremely severe psoriasis*”/“*extreme discomfort/extreme pain*” as well as a Likert‐scale (−3 to +3) to rate patients' preference for topical Cal/BD foam treatment with or without AFL pretreatment.

#### 2.4.3 Optical coherence tomography imaging

OCT was used to guide laser settings based on nail plate thickness, determine if the nail bed was harmed by AFL, and evaluate the subclinical treatment response to support clinical efficacy assessments using 15 predefined image features extracted from the literature.^40^

OCT is a noninvasive in vivo imaging technique that uses laser interferometry to generate high‐resolution images down to a depth of 2 mm. In this study, a swept‐source OCT (Vivosight Rx, Michelson Diagnostics) was used. First, single‐frame B‐scans were captured at baseline and at the EOT visit to measure nail plate thickness. Second, four digits with the highest N‐NAIL score at baseline were scanned before and after AFL treatment. These full scans were repeated at Week 6, Week 12, and EOT. All scans were captured by an experienced project nurse and anonymized before remote analysis by a blinded expert reader (VDM) using a predefined list of previously reported PND features;^40^ images were assessed qualitatively (yes/no) for periungual, nail plate, nail bed, and vascular changes.

#### 2.4.4 Smartphone app‐based safety monitoring

**FIGURE 2:**
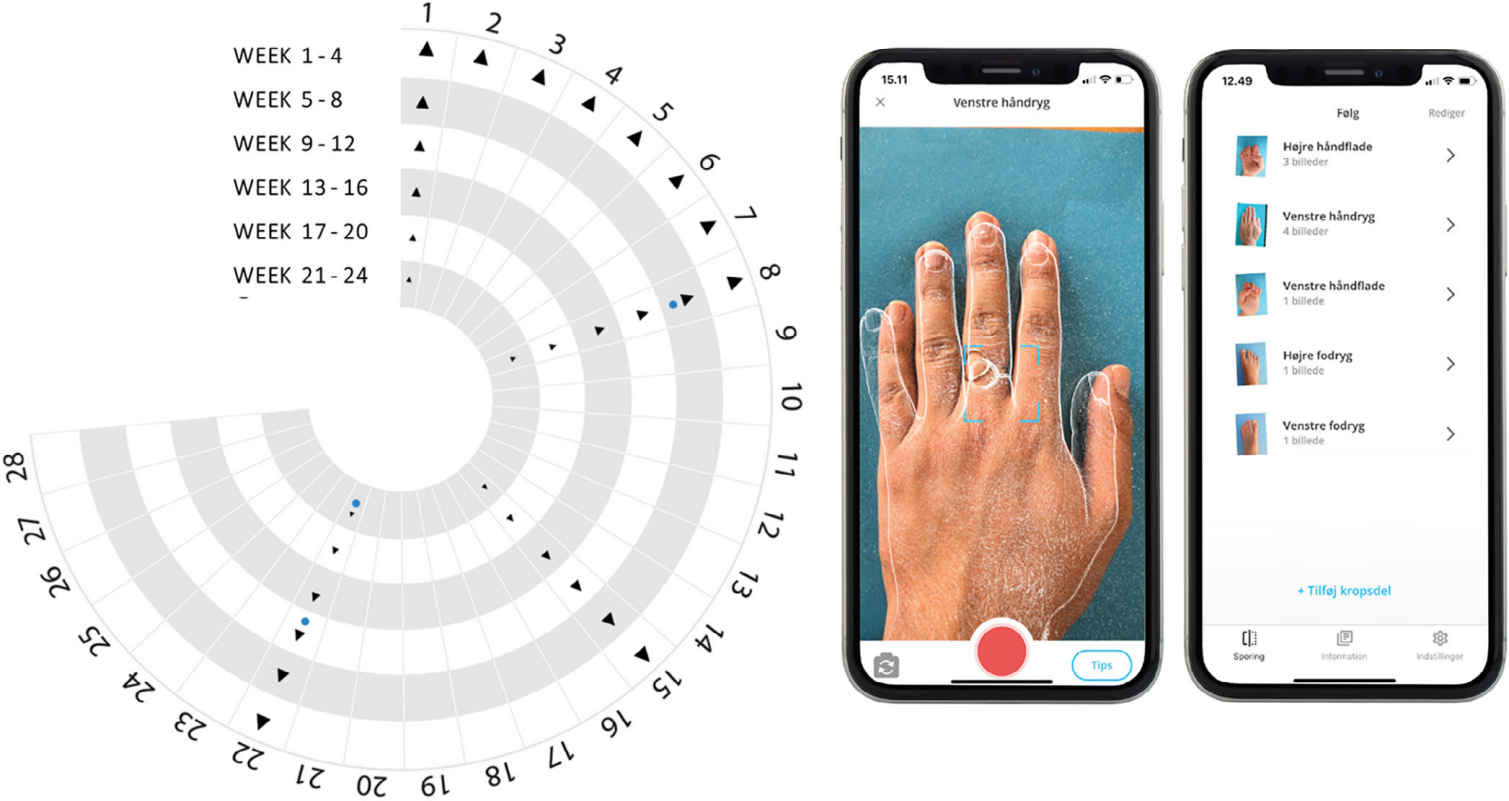
Schematic overview of safety monitoring in a hybrid trial on psoriatic nail disease using Calcipotriol Betamethasone Dipropionate (Cal/BD) aerosol foam treatment with and without laser pretreatment using a smartphone app. Patients were asked to collect image data for 24 weeks at predefined time points (left) using a customized smartphone application (right) that creates transparent overlay images of anatomical landmarks to standardize photographs. Patients received notifications to remind them to capture photographs (black triangle) and attend their *in‐clinic* follow‐up visits (blue dot)

Patients were instructed to download a smartphone app (Imagine Skin Tracker, Omhu, LEO Innovation Lab) to obtain standardized images of their treatment areas for remote monitoring of potential side effects and local skin reactions. To allow for a remote safety check‐up, patients were requested to use the smartphone app daily for the first week and weekly until EOT to take pictures of affected hands and feet (Figures 2 and S1). For each check‐up, patients captured a total of six photographs: two of each hand in two different positions and one of each foot. Using a web application, the treating physician assessed smartphone pictures *in‐clinic* for local skin reactions (LSR 0 = no evidence of irritation; I = minimal erythema; II = definite erythema; III = erythema and papules; IV = erythema and papules and visible swelling; V = vesicular eruption; VI = strong reaction spreading beyond treatment area) using a previously published grading scale as well as for the presence of other skin and nail reactions.^44^ In case of safety concerns, patients were contacted for an additional *in‐clinic* follow‐up visit.

### 2.5 Data management and statistics

Study data were collected and managed using REDCap (Research Electronic Data Capture) electronic data capture tools hosted at Region Hovedstaden (Capital Region),^45^, ^46^ and analyzed using R in R Studio version 1.2.5. Summary measures are presented as medians and inter‐quartile ranges (IQR) where applicable. Clinical and subclinical treatment response were described as mean percentage changes, tested for normality using Shapiro–Wilk normality test, evaluated using Bonferroni‐corrected multiple paired‐*t* tests, and visualized as boxplots. Pearson correlation measures (r) were calculated and presented as a correlation matrix. *p*‐values less than 0.05 were considered significant.

## 3 RESULTS

A total of 11 patients (age 52 IQR 22; 27% female) with 144 affected nails with a mean N‐NAIL baseline score of 63 (IQR 39.5) were recruited via social media (Facebook [*n* = 8], Danish Psoriasis Association [*n* = 2]), and in our outpatient clinic (*n* = 1). All patients had previously been treated with either topical treatments other than Cal/BD foam (*n* = 11), methotrexate (*n* = 5), intralesional injection (*n* = 2), or PUVA (*n* = 1). Ten patients completed the trial after 24 weeks of treatment; one patient attended their EOT visit at W12 due to his psoriasis requiring biological treatment. A total of 71 finger‐ and 73 toenails were included (*n* = 144), of which 70 nails (48.6%) received AFL. At baseline, there was no significant difference between the two treatment groups in terms of disease severity (*p* = 0.596).

All patients showed signs of psoriasis (PASI: baseline 1.3 [IQR 2.95]; EOT 1.3 [IQR 3.45]), two patients had previously been diagnosed with psoriatic arthritis. Methotrexate treatment and background medication for comorbidities (hypertension [*n* = 3], depression [*n* = 2], erectile dysfunction [*n* = 2], arthralgia [*n* = 1], neuralgia [*n* = 1], hypercholesterolemia [*n* = 1], benign prostatic hyperplasia [*n* = 1], estrogen deficiency [*n* = 1]) were continued throughout the treatment period.

Patients used a median of 83.1 g (IQR 44.2 g) of Cal/BD foam during the trial, corresponding to 5.7 g (IQR 3.0 g) per nail. The total amount patients applied per nail ranged between 4.0 and 21.0 g over 24 weeks.^47^ Figure S2 shows that there were no statistically significant correlations between the amount of Cal/BD foam per nail and reduction in clinical severity, OCT‐assessed signs of PND, or local skin reactions.

### 3.1 Clinical and OCT‐assessed efficacy

Treatment efficacy was assessed *in‐clinic* by the treating physician using the clinical N‐NAIL score as well as *remotely* using clinical photographs for clinical NAPSI scoring and subclinical OCT image evaluation by a blinded dermatologist.

**FIGURE 3:**
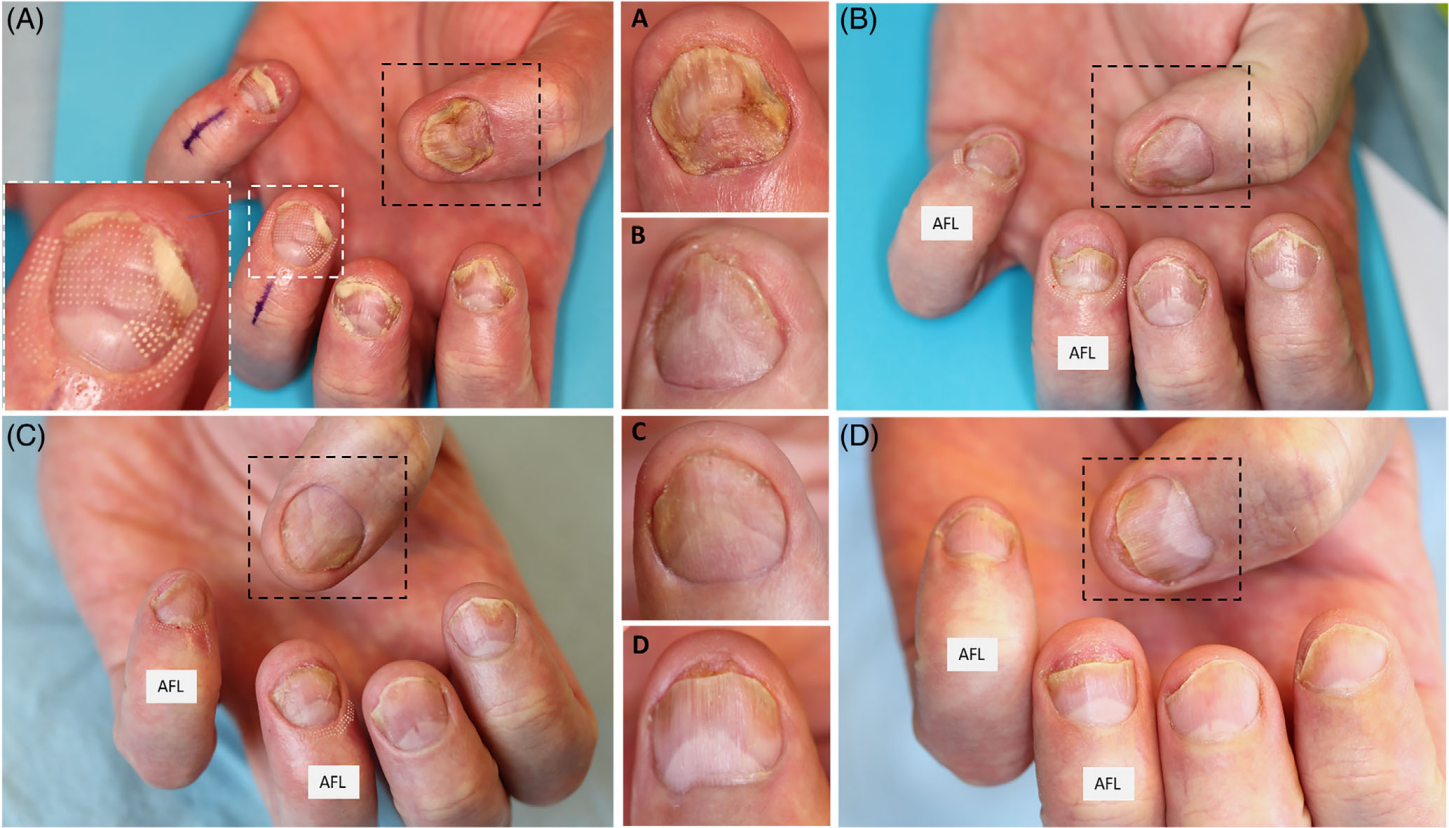
Representative photographs of psoriatic nails shown at baseline (A) and following Calcipotriol/Betamethasone Dipropionate (Cal/BD) aerosol foam treatment with and without ablative fractional laser (AFL) treatment after 6 week (B), 12 week (C), and 24 weeks (D) showing visible improvement of the proximal nail unit

After 24 weeks of Cal/BD treatment, there was a clinically significant treatment effect (median N‐NAIL baseline: 62, EOT: 15, *p* = 0.001) as shown in the representative photographs in Figure 3, with no significant differences between finger‐ and toenails (*p* = 0.35). The median scores of the clinical PND assessment for both treatment groups, as well as for finger‐ and toenails are summarized in Table 3. AFL pretreatment had no statistically significant effect on the Cal/BD foam treatment response (see Figure S3) but showed a positive trend in clinical improvement (+19.3% reduction in median N‐NAIL score, *p* = 0.62; +20.0% reduction in median NAPSI score, *p* = 0.45).

**TABLE 3:** Clinical ratings based on in‐clinic unblinded N‐NAIL or photograph‐based remote blinded NAPSI of psoriatic nails treated with Cal/BD foam, either as monotherapy or in conjunction with ablative fractional laser

		Cal/BD treated nails		Cal/BD + AFL		Cal/BD only		Fingernails		Toenails	
Baseline	N‐NAIL (median)	62 (42.5)		33 (21.5)		29 (21)		20 (24)		25 (28)	
6 weeks		35 (32.5)	−44%	15 (18)	−55%	20 (14)	−31%	15 (16.5)	−25%	20 (20.5)	−20%
12 weeks		25 (40)	−60%	13 (18)	−61%	13 (18)	−55%	16 (17.5)	−20%	16 (20.5)	−36%
EOT		15 (36)	−76%	5 (18)	−85%	10 (16.5)	−66%	5 (16.5)	−75%	8 (19)	−68%
Baseline	NAPSI (median)	80 (63)		40 (27.5)		40 (32)		36 (40)		45 (34.5)	
6 weeks		60 (71)	−25%	29 (38)	−28%	31 (32)	−23%	26 (40.5)	−28%	29 (36)	−36%
12 weeks		41 (67)	−49%	17 (35.5)	−58%	25 (28)	−38%	18 (32)	−50%	23 (38)	−49%
EOT		26 (43)	−68%	9 (23.5)	−78%	17 (20.5)	−58%	13 (27)	−64%	17 (21)	−62%

*Note*: Results are presented as medians with interquartile range in brackets, and the median reduction of the median as percentage.

Abbreviations: AFL, ablative fractional laser; Cal/BD, calcipotriol betamethasone dipropionate; EOT, end of trial; NAPSI, nail psoriasis severity index; N‐NAIL, Nijmegen‐nail psoriasis activity index tool.

**FIGURE 4:**
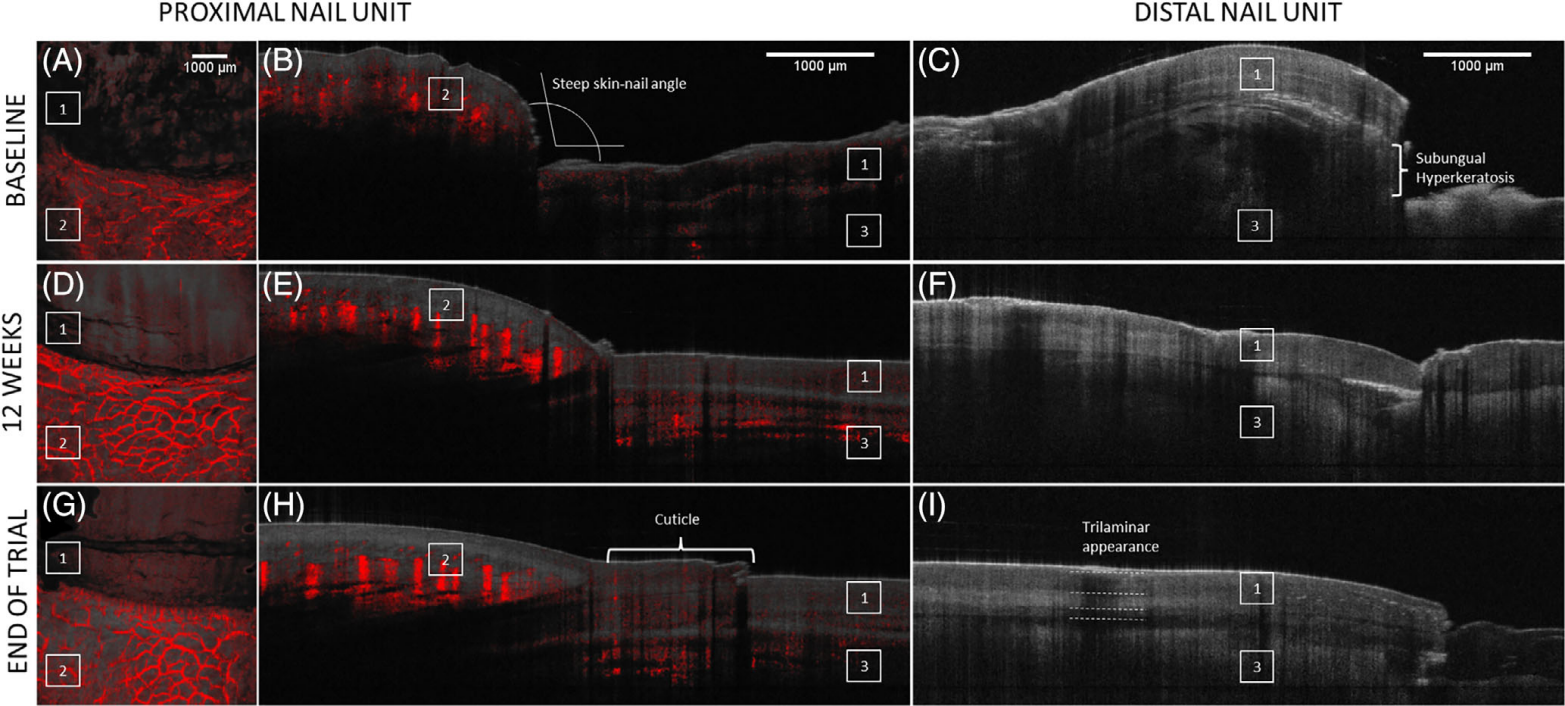
Optical coherence tomography (OCT) images of the proximal and distal part of the nail unit at baseline (A–C), week 12 follow‐up (D–F), and the end of treatment visit in week 24 (G–I) showing a gradual improvement of the psoriatic nail unit in response to Calcipotriol/Betamethasone dipropionate foam treatment. En‐face dynamic OCT images (A, D, G) show normalization of the nail fold microvasculature (red) whereas structural OCT (gray) shows changes in skin‐nail angle (B) and cuticle regrowth (H), reduction of subungual hyperkeratosis (C) and reappearance of trilaminar nail plate structure (I)

The overall decrease in subclinical OCT features showed a strong correlation with clinical NAPSI reduction in PND symptoms (at EOT *r* = 0.96, *p* < 0.0001). In all patients, OCT was able to visualize a progressive reduction in the, at baseline, increased density of blood vessels extending superficially into the proximal nail fold. The overall prevalence of psoriatic OCT features, summarized in Table 4, dropped significantly in response to treatment with Cal/BD foam after 6 (−13%, *p* = 0.006), 12 (−27%, *p* = 0.002), and 24 weeks (−42%, *p* < 0.0001). Figure 4 illustrates the gradual normalization of the nail unit as a response to Cal/BD treatment. Further, there was no significant difference in reduction of total prevalence of psoriatic OCT features between Cal/BD and Cal/BD + AFL at baseline (*p* = 0.596), week 6 (*p* = 0.07), week 12 (*p* = 0.158), or EOT (*p* = 0.888) but an on average higher reduction in the Cal/BD + AFL treated nails (week 6: −14%, week 12: −29%, EoT: −46%). Cal/BD + AFL treated nails showed a notably higher reduction in the presence of a “*ragged skin surface of the proximal nail fold*” (−52% vs. −82%), the “*epidermal thickening of the proximal nail fold*” (−48% vs. −82%), and a “*steep skin‐nail angle*” (−48% vs. −82%).

**TABLE 4:** Overview of optical coherence tomography features and their prevalence in psoriatic nails at baseline, after 6 weeks, after 12 weeks, and after 24 weeks (end of trial) of daily calcipotriol/betamethasone dipropionate aerosol foam

	Baseline			Week 6			Week 12			End of trial (Week 24)		
	Total	Cal/BD	AFL + Cal/BD	Total	Cal/BD	AFL + Cal/BD	Total	Cal/BD	AFL + Cal/BD	Total	Cal/BD	AFL + Cal/BD
Ragged skin surface of the PNF	76%	65%	86%	47% (−29%)	35% (−30%)	59% (−27%)	22% (−53%)	22% (−43%)	23% (−64%)	12% (−63%)	13% (−52%)	5% (−82%)
Epidermal thickening of the PNF	73%	61%	86%	44% (−29%)	35% (−26%)	55% (−32%)	29% (−44%)	22% (−39%)	36% (−50%)	12% (−61%)	13% (−48%)	5% (−82%)
Steep skin‐nail angle	67%	52%	82%	42% (−24%)	35% (−17%)	50% (−32%)	7% (−60%)	4% (−48%)	9% (−50%)	3% (−64%)	4% (−48%)	0% (−82%)
Dilated vessels in a haphazard orientation in PNF	89%	83%	95%	80% (−9%)	70% (−13%)	91% (−5%)	76% (−13%)	65% (−17%)	86% (−9%)	45% (−43%)	48% (−35%)	55% (41%)
Increased density of blood vessels extending superficially in PNF	89%	83%	95%	80% (−9%)	70% (−13%)	91% (−5%)	76% (−13%)	65% (−17%)	86% (−9%)	45% (−43%)	48% (−35%)	55% (−41%)
Wavy, irregular, and rough nail plate surface	96%	96%	95%	80% (−16%)	83% (−13%)	77% (−18%)	58% (−38%)	48% (−48%)	68% (−27%)	27% (−68%)	13% (−83%)	27% (−68%)
Superficial fissuring of the nail plate	100%	100%	100%	98% (−2%)	96% (−4%)	100% (–)	93% (−7%)	91% (−9%)	95% (−5%)	79% (−21%)	78% (−22%)	77% (−23%)
Pitting	71%	65%	77%	62% (−9%)	57% (−9%)	68% (−9%)	40% (−31%)	52% (−13%)	27% (−50%)	27% (−44%)	26% (−39%)	23% (−50%)
Nail plate thickening	71%	61%	82%	47% (−24%)	39% (−22%)	55% (−27%)	29% (−42%)	17% (−43%)	41% (−41%)	18% (−53%)	4% (−57%)	23% (−59%)
Loss of trilaminar appearance	100%	100%	100%	100% (−)	100% (−)	100% (−)	93% (−7%)	91% (−9%)	95% (−5%)	91% (−9%)	91% (−9%)	86% (−14%)
Leukonychia	29%	30%	27%	13% (−16%)	17% (−13%)	9% (−18%)	7% (−22%)	9% (−22%)	5% (−23%)	0% (−29%)	0% (−30%)	0% (−27%)
Hyperreflective spots in the nail plate	11%	13%	9%	11% (−)	13% (−)	9% (−)	7% (−4%)	9% (−4%)	5% (−5%)	6% (−5%)	9% (−4%)	5% (−5%)
Onycholysis	98%	100%	95%	96% (−2%)	100% (−)	91% (−5%)	93% (−4%)	96% (−4%)	91% (−5%)	88% (−10%)	96% (−4%)	82% (−14%)
Subungual hyperkeratosis	91%	91%	91%	73% (−18%)	78% (−13%)	68% (−23%)	67% (−24%)	74% (−17%)	59% (−32%)	64% (−27%)	57% (−35%)	50% (−41%)
Abnormalities of the deep nail bed	76%	74%	77%	67% (−9%)	70% (−4%)	64% (−14%)	33% (−42%)	26% (−48%)	41% (−36%)	21% (−54%)	13% (−61%)	18% (−59%)
Total feature prevalence	76%	72%	80%	63% (−13%)	60% (−12%)	66% (−14%)	49% (−27%)	46% (−26%)	51% (−29%)	34% (−42%)	35% (−37%)	34% (−46%)

*Note*: The change in prevalence as mean percentage change in comparison to baseline is presented in brackets.

Abbreviations: AFL, ablative fractional laser; Cal/BD, calcipotriol betamethasone dipropionate; PNF, proximal nail fold.

### 3.2 Patient‐reported outcome measures

The improvement in PROMs, collected *in‐clinic*, corresponded to the clinically and OCT‐assessed efficacy. The nail‐disease adapted mDLQI score dropped 60% over 24 weeks from a baseline score of 5 to an EOT score of 2. Similarly, patients rated the severity of their PND on a 10‐point scale as 7 at baseline, steadily improving reaching 4.5 in Week 6, 3 in Week 12, and 2 at the end of the trial (reduction of 71%). Correspondingly, patients considered the treatment increasingly more efficacious, with a score of 3.5 at Week 6, 5 in Week 12, and 6.5 at the EOT in Week 24 (+85.7%). The initial discomfort due to Cal/BD foam and AFL, which patients considered to be tolerable, dropped from 3 in Week 6 to 1.5 in Week 12 and lastly 1 in Week 24 (−66.7%). Patients showed no preference for Cal/BD foam with or without laser pretreatment. All PROMs are summarized in Table 5.

**TABLE 5:** Patient‐reported outcomes reported during Cal/BD treatment with/without fractional ablative CO_2_ laser treatment

Patient‐reported outcome measures	Time point	Median	IQR	Change from baseline^a^
mDLQI (0–30)	Baseline	5.0	4.0	–
	Week 6	3.0	2.5	−40.0%
	Week 12	2.0	1.5	−60.0%
	End of trial	2.0	1.75	−60.0%
Patient‐assessed nail disease severity (0–10)	Baseline	7.0	1.75	–
	Week 6	4.5	1.75	−35.7%
	Week 12	3.0	4.75	−57.1%
	End of trial	2.0	2.75	−71.4%
Patient‐assessed treatment efficacy (0–10)	Baseline	0.0	0.0	–
	Week 6	3.5	4.5	–
	Week 12	5.0	3.75	+42.9%
	End of trial	6.5	1.75	+85.7%
Treatment‐related discomfort (0–10)	Baseline	0.0	0.0	–
	Week 6	3.0	3.0	–
	Week 12	1.5	2.75	−50%
	End of trial	1.0	1.75	−66.7%
Treatment preference (−3–+3) (−3: strong preference for Cal/BD; +3: strong preference for Cal/BD and AFL)	Baseline	0.0	0.0	–
	Week 6	0.0	0.75	–
	Week 12	0.0	1.5	–
	End of trial	0.0	0.0	–

*Abbreviations: AFL, ablative fractional laser; Cal/BD, calcipotriol betamethasone dipropionate; IQR, interquartile range; mDLQI, modified dermatology life quality index*.

^*a*^ *End of trial: 24 weeks*.

### 3.3 Safety

The safety and tolerability of Cal/BD and AFL were assessed in‐clinic based on physical examination at follow‐up visits and smartphone photographs obtained remotely by the patient. Out of 2176 images collected using the smartphone application over the course of 24 weeks, 95% (*n* = 2073) were deemed acceptable for safety monitoring.

*In‐clinic* evaluation of smartphone images by the treating physician using a web application showed erythema (minimal: 44%, definite: 31%), swelling (28%), crusting (27%), distal skin peeling (20%), onychoschizia (9%), hyperpigmentation (7%), proximal skin peeling (5%), erosion (2%), flaking (2%), signs of nail fold infection (1%), and fissures (1%). Distal skin peeling, erosions, paronychia, and fissures were only seen on toes. In two instances, patients were asked to attend *in‐clinic* follow‐up visits for further assessment of suspected paronychia and delayed wound healing.

In addition to photograph‐assessed local skin reactions and nail changes, the following adverse events were reported following *in‐clinic* physical examination: acute paronychia (*n* = 1), onychocryptosis (*n* = 1), persistent post‐laser erythema (*n* = 1), transient application site pain (*n* = 1), proximal interphalangeal joint pain (*n* = 1), and transient reduction in grip strength (*n* = 1).

Lastly, OCT was used to assess if AFL treatment caused any damage to the nail bed. While the microporation did not reach the nail bed in any of our patients, short‐lived (<10 s) painful sensations immediately after AFL treatment were reported. With a median OCT‐measured nail plate thickness of 713 μm (IQR 338 μm) and micropore depth of 181 μm (IQR 84.5 μm), the mean degree of poration was 34% (IQR 22%); the highest degree of OCT‐assessed poration was 83%. Figure S4 shows OCT images of psoriatic nail units immediately after baseline AFL treatment.

## 4 DISCUSSION

This first hybrid trial on PND provided new evidence on the long‐term treatment with Cal/BD foam, the feasibility of OCT‐guided AFL pretreatment of the nail unit, and the value of smartphone‐assisted remote patient monitoring to reduce the number of in‐clinic visits. By combining in‐clinic, OCT, and photo‐based safety monitoring, we piloted new safety and efficacy evaluation concepts by taking advantage of noninvasive imaging and remote assessments to change patient care. Our findings suggest that Cal/BD foam, both with and without pretreatment, could be an efficacious and tolerable long‐term treatment option for dermatologists managing patients with PND, including those already receiving methotrexate.

The far‐reaching impact of COVID‐19 has forced health care practitioners, researchers, and patients to become more tech‐savvy and embrace the use of digital media. While safety assessments in conventional trials are limited to *in‐clinic* assessments, partial decentralization allowed patients in this trial to collect a median of 191 smartphone images that represent a valuable resource for the comprehensive evaluation and photo documentation of this new treatment approach. Although the high rate of side effects may be due to the drug‐device combination, the use of high‐frequency *remote* monitoring in a long‐term treatment trial puts previous reports on AE into perspective (see Table 1). Based on our early‐adopter experience, virtual monitoring of PND treatment can increase safety in clinical trials but will likely lead to higher incidence of side effects even for well‐established treatments.

While Cal/BD foam is one of the staple treatments for cutaneous psoriasis,^48^ only one RCT has investigated this product for PND, reporting a reduction in NAPSI of 44% after 12 weeks of daily application.^4^ The notably higher efficacy rates in our study after 24 weeks of treatment (−76% N‐NAIL, −68% NAPSI) with comparable safety profile and improvement in PROMs indicate a favorable risk–benefit ratio for prolonged treatment with Cal/BD foam. While the currently available evidence may be insufficient for recommendations, more investigations are underway to determine the role of Cal/BD foam in the management of PND.^49^, ^50^

To date, two RCTs have explored the use of AFL pretreatment for laser‐assisted ungual delivery (LAUD) with daily application of Tazarotene gel and Cal/BD gel for 3 months. Although both studies demonstrated side effects similar to ours, their efficacy rate of −48% mNAPSI and −34% NAPSI, respectively, suggesting that treatment duration and dosage form are key factors in topical PND treatment. The treatment effect in the Tazarotene gel trial, however, was significantly better in patients receiving AFL‐pretreatment, which may indicate that gel formulations are better suited for LAUD.^3^, ^33^ Further pre‐clinical and clinical investigations on the compatibility of supersaturated foam with laser‐assisted drug delivery are warranted to determine if and to what extent LAUD can increase the bioavailability of Cal/BD foam in PND.

In the context of laser treatment of nail tissue, OCT proved to be helpful in determining ablation depth and was therefore used to guide AFL pretreatment.^52^, ^53^ By noninvasively measuring the thickness of the nail plate, we were able to safely adjust the pulse energy to a level that would spare the nail bed. In this trial, OCT was also used for blinded *remote* evaluations of subclinical PND treatment response, which correlated highly with *in‐clinic* efficacy assessments. Subclinical distal “*onycholysis*” as well as features that lack a clinical correlate such as “*loss of trilaminar appearance*” may, however, have contributed to false‐positive ratings of ongoing psoriatic activity which underscores the importance of a longer follow‐up and a more granular OCT image analysis. The growing evidence on OCT imaging of PND calls for the development of a validated scoring system to promote its standardized use in clinical practice and facilitate quantitative OCT assessments in research.

### 4.1 Limitations

Given the proof‐of‐concept nature of this study, several confounding factors could not be accounted for. This included handedness, occupation, and daily activities and their potential impact on the disease course and treatment response. Further, in‐depth comparisons between psoriatic finger‐ and toenails were limited by the study design and population. Larger RCTs with sufficiently powered subgroup analyses are needed to address these potential confounders and determine a more nuanced safety and efficacy profile for LAUD and Cal/BD foam treatment.

## 5 CONCLUSION

The results of this exploratory hybrid trial suggest that the clinical efficacy and safety profile of Cal/BD foam, both with and without laser pretreatment, justify its use in suitable long‐term treatment for patients with PND. Supported by a pronounced improvement in PROMs and subclinical image markers, larger studies with longer follow‐up are warranted to determine if the enhanced delivery of Cal/BD foam should be recommended for patients not responding to other topical or systemic treatments.

## AUTHOR CONTRIBUTIONS

Study conception and design: Vinzent Kevin Ortner, Victor Desmond Mandel, Kresten Skak, John Robert Zibert, Christine Sofie Krohn Fuchs, Peter Alshede Philipsen, Merete Haedersdal. Acquisition of data: Vinzent Kevin Ortner, Christoffer Valdemar Nissen, Mélanie Bourlioux, Victor Desmond Mandel, Merete Haedersdal. Analysis and interpretation of data: Vinzent Kevin Ortner, Victor Desmond Mandel, Peter Alshede Philipsen, Merete Haedersdal. Drafting of manuscript: Vinzent Kevin Ortner, Merete Haedersdal. Critical revision: Vinzent Kevin Ortner, Victor Desmond Mandel, Kresten Skak, John Robert Zibert, Mélanie Bourlioux, Christoffer Valdemar Nissen, Christine Sofie Krohn Fuchs, Peter Alshede Philipsen, Merete Haedersdal.

## FUNDING INFORMATION

Merete Haedersdal has received research grants from Innovation Fund Denmark and LEO Pharma A/S.

## CONFLICT OF INTEREST

Vinzent Kevin Ortner, Kresten Skak, Mélanie Bourlioux, John Robert Zibert, and Merete Haedersdal have previously been employed at LEO Pharma A/S.

## Supporting information


**Supplementary Table S1:** Modified dermatological life quality index (mDLQI) for psoriatic nail disease.

**Supplementary Figure S1:** User instructions for virtual safety monitoring to ensure a neutral background (A) is selected, images are captured at a standardized angle in parallel to the treatment area (B), standardized digit placement within the ghost image outline (C), correct distance to ensure sharp images (D), the area of interest covers 90% of photograph (E), and to follow the guidance on standardized hand positioning for complete view of all digits (F).

**Supplementary Figure S2:** Visualization of the relationship between clinical (NAPSI) and subclinical (OCT) changes, dosage, and local skin reactions using Pearson correlation testing. The size of the symbol corresponds to the significance of the correlation and is marked with an asterisk for significance levels of *p* < 0.05. The color indicates the strength of correlation, ranging from r = 1 (dark blue, perfect positive correlation) to r = 0 (white, no correlation) to r = −1 (dark red, perfect negative/inverse correlation).

**Supplementary Figure S3:** Boxplot comparison of Calcipotriol Betamethasone Dipropionate (Cal/BD) aerosol foam treatment of psoriatic nail disease with or without ablative fractional laser (AFL) pretreatment. (Cal/BD vs. Cal/BD + AFL) presented as panels with color‐coded boxplots (blue: with AFL, yellow: without AFL) visualizing a gradual reduction in clinical severity scored using the Nail Psoriasis Severity Index (NAPSI) and the Nijmegen‐Nail psoriasis Activity Index tool (N‐NAIL).

**Supplementary figure S4:** Optical coherence tomography images of psoriatic nail plate and folds immediately after ablative fractional laser treatment. Laser‐tissue interactions are marked in red.

## Data Availability

Data available on request due to privacy/ethical restrictions.
