# Supplementary material for: Investigating the efficacy and safety of calcipotriol/betamethasone dipropionate foam and laser microporation for psoriatic nail disease—A hybrid trial using a smartphone application, optical coherence tomography, and patient‐reported outcome measures
Source: Dermatol Ther. 2022 Nov 23;35(12):e15965. doi: 10.1111/dth.15965 (PMC10078349; doi:10.1111/dth.15965)
Supplement: Supplementary file 1 — Supplementary Table S1: Modified dermatological life quality index (mDLQI) for psoriatic nail disease. [file DTH-35-0-s003.docx]

**Dermatology life quality index**(modified for psoriatic nail disease)

Patient ID:

Visit:

|  | Very much | A lot | A little | Not at all |
| --- | --- | --- | --- | --- |
| Over the last week, how itchy, sore, painful or stinging have your nails and surrounding skin been? |  |  |  |  |
| Over the last week, how embarrassed or self-conscious have you been because of your nails? |  |  |  |  |
| Over the last week, how much have your nails interfered with you going shopping or looking after your home or yard? |  |  |  |  |
| Over the last week, how much have your nails influenced the clothes you wear? |  |  |  |  |
| Over the last week, how much have your nails affected any social or leisure activities? |  |  |  |  |
| Over the last week, how much have your nails made it difficult for you to do any sport? |  |  |  |  |
| Over the last week, how much have your nails created problems with your partner or any of your close friends or relatives? |  |  |  |  |
| Over the last week, how much have your nails caused any sexual difficulties? |  |  |  |  |
| Over the last week, how much of a problem has the treatment for your nails been, for example by making your home messy, or by taking up time? |  |  |  |  |
|  | **Yes** | **No** |  |  |
| Ove the last week, have your nails prevented you from working or studying? |  |  |  |  |

Please check you have answered every question. Thank you.

Date: __-__-__ Signature:_____________________

Very much: 3
A lot: 2
A little: 1
Not at all: 0
Yes: 3
No: 0
