# Supplementary figures and images for: Investigating the efficacy and safety of calcipotriol/betamethasone dipropionate foam and laser microporation for psoriatic nail disease—A hybrid trial using a smartphone application, optical coherence tomography, and patient‐reported outcome measures
Source: Dermatol Ther. 2022 Nov 23;35(12):e15965. doi: 10.1111/dth.15965 (PMC10078349; doi:10.1111/dth.15965)

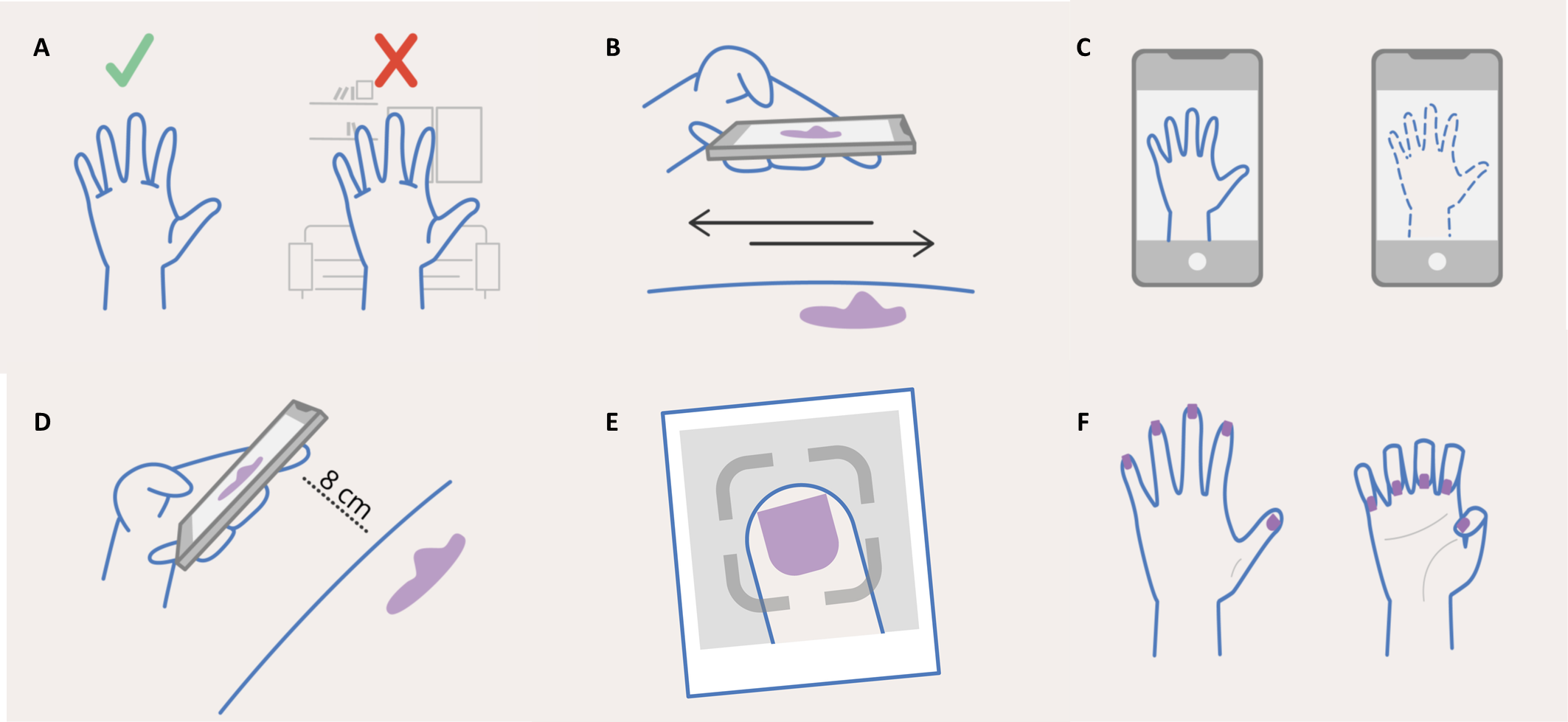

Supplement: Supplementary file 2 — Supplementary Figure S1: User instructions for virtual safety monitoring to ensure a neutral background (A) is selected, images are captured at a standardized angle in parallel to the treatment area (B), standardized digit placement within the ghost image outline (C), correct distance to ensure sharp images (D), the area of interest covers 90% of photograph (E), and to follow the guidance on standardized hand positioning for complete view of all digits (F). [file DTH-35-0-s004.png]

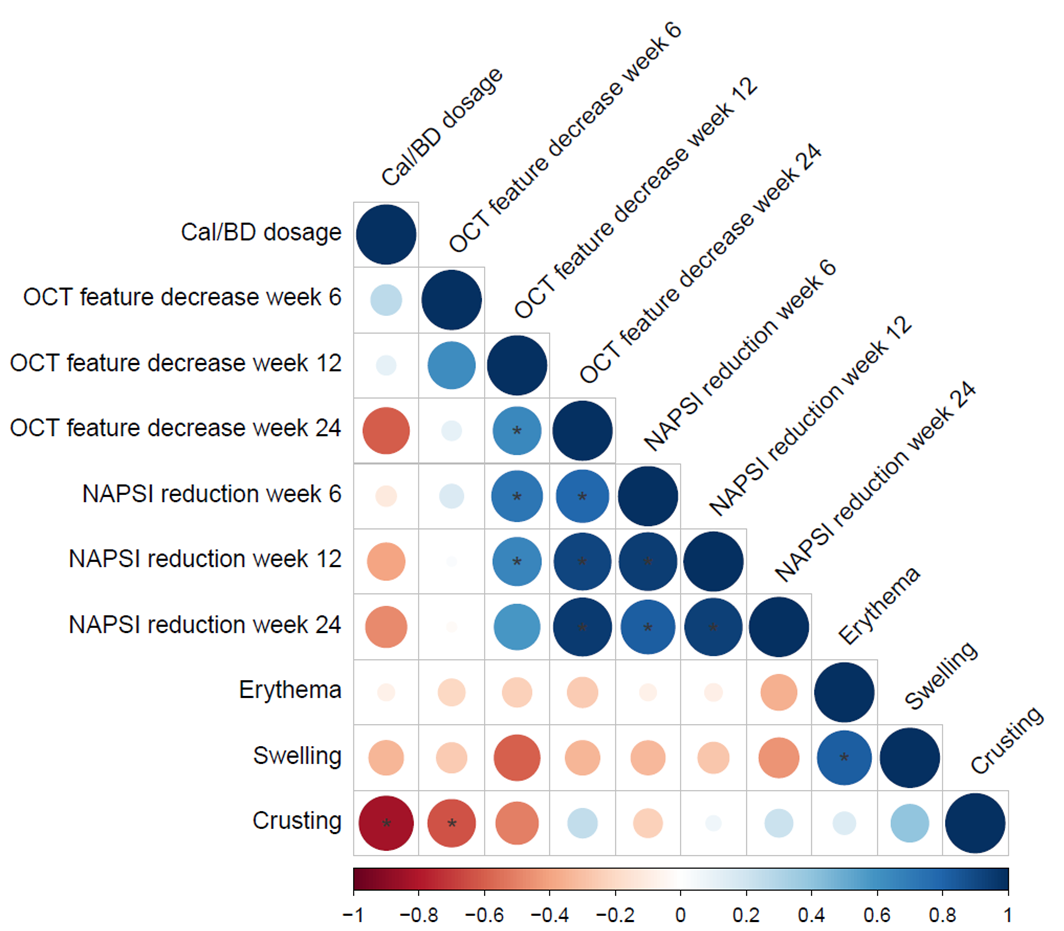

Supplement: Supplementary file 3 — Supplementary Figure S2: Visualization of the relationship between clinical (NAPSI) and subclinical (OCT) changes, dosage, and local skin reactions using Pearson correlation testing. The size of the symbol corresponds to the significance of the correlation and is marked with an asterisk for significance levels of p < 0.05. The color indicates the strength of correlation, ranging from r = 1 (dark blue, perfect positive correlation) to r = 0 (white, no correlation) to r = −1 (dark red, perfect negative/inverse correlation). [file DTH-35-0-s002.png]

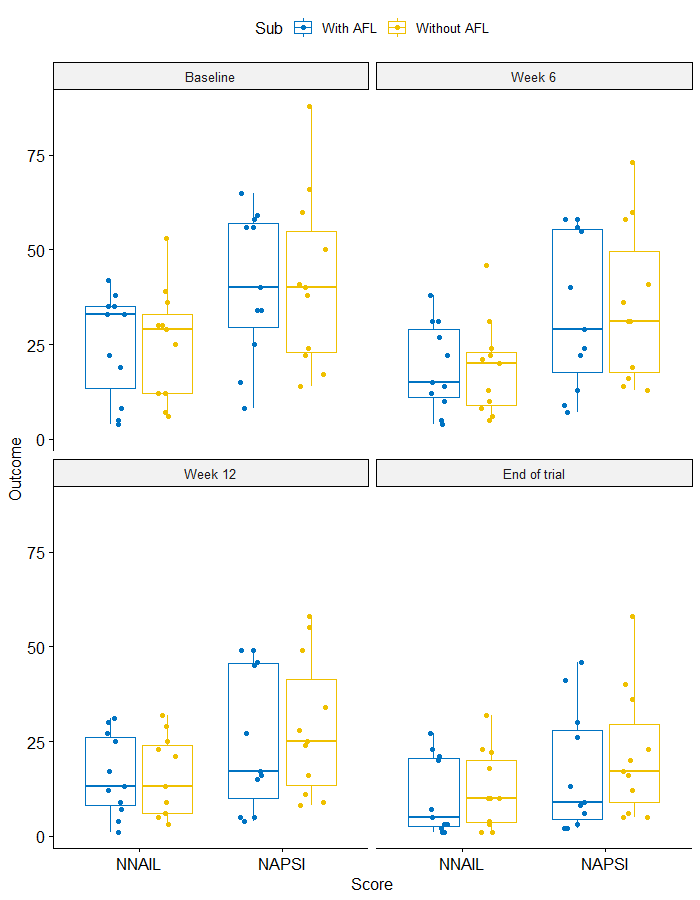

Supplement: Supplementary file 4 — Supplementary Figure S3: Boxplot comparison of Calcipotriol Betamethasone Dipropionate (Cal/BD) aerosol foam treatment of psoriatic nail disease with or without ablative fractional laser (AFL) pretreatment. (Cal/BD vs. Cal/BD + AFL) presented as panels with color‐coded boxplots (blue: with AFL, yellow: without AFL) visualizing a gradual reduction in clinical severity scored using the Nail Psoriasis Severity Index (NAPSI) and the Nijmegen‐Nail psoriasis Activity Index tool (N‐NAIL). [file DTH-35-0-s001.png]

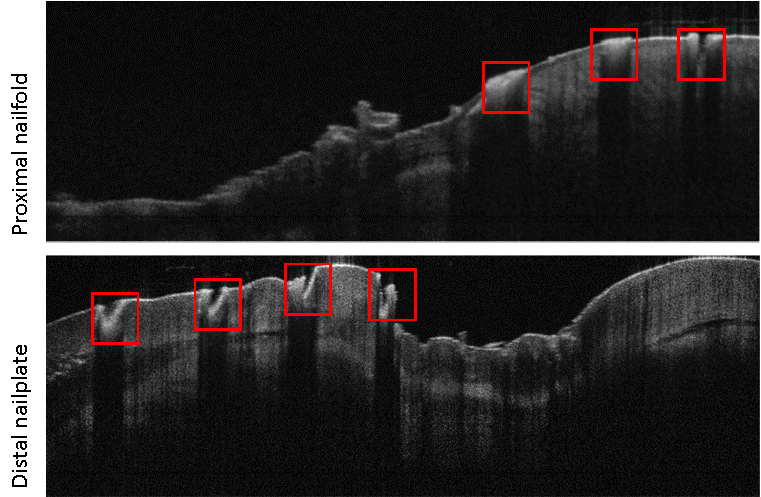

Supplement: Supplementary file 5 — Supplementary figure S4: Optical coherence tomography images of psoriatic nail plate and folds immediately after ablative fractional laser treatment. Laser‐tissue interactions are marked in red. [file DTH-35-0-s005.png]
